# Costs of human papillomavirus vaccine delivery in low- and middle-income countries: A systematic review

**DOI:** 10.1016/j.vaccine.2024.01.094

**Published:** 2024-02-27

**Authors:** Rose Slavkovsky, Emily Callen, Clint Pecenka, Mercy Mvundura

**Affiliations:** a Center for Vaccine Innovation and Access, PATH, Seattle, WA, USA; b Comparative Health Outcomes, Policy, and Economics (CHOICE) Institute, University of Washington, Seattle, WA, USA; c Medical Devices and Health Technologies, PATH, Seattle, WA, USA

**Keywords:** Costs and cost analysis, Human papillomavirus vaccine, Systematic review, Immunization delivery, Immunization costing, Low- and middle-income countries

## Abstract

Vaccines to protect against human papillomavirus (HPV) infection are recommended for all adolescents by the World Health Organization (WHO) and are primarily delivered in school-based settings. This systematic review aims to summarize the available evidence on the cost of HPV vaccine delivery in low- and middle-income countries (LMICs). This updated evidence is eminent given recent global efforts to revitalize HPV vaccine delivery following the COVID-19 pandemic and can be used to inform planning for program sustainability.

We carried out a systematic review of published literature reporting the costs of HPV vaccine delivery in LMICs published between 2005 and 2023. Eligibility criteria were developed using the Population, Intervention, Comparator, Outcome (PICO) framework, and studies that reported primary costing data and unit costs of HPV vaccine delivery were included. From the included studies, we extracted data such as phase of HPV vaccine implementation when costing was done, delivery strategy, and unit costs. Unit costs were converted into 2022 US$ for comparability. All included studies underwent critical appraisal using an adapted framework including Consolidated Health Economics Evaluation Reporting Standards criteria, the WHO-led consensus statement on vaccine delivery costing, and other frameworks.

Our research identified 226 records, of which 15 met our inclusion criteria. Most studies (64 %) were carried out in African countries and during HPV vaccine pilots or demonstrations (60 %). Vaccine delivery cost ranged from $0.31 to $24.07 per dose for financial costs and $1.48 to $48.70 per dose for economic costs. The critical appraisal showed that most studies did not describe the uncertainty of reported delivery cost.

Our systematic review evidence suggests that HPV vaccine delivery costs vary widely depending on country and stage of implementation when costing was done. Areas for further research include costing when programs are beyond the introduction phase and in LMICs outside of Africa.

## 1 Introduction

Cervical cancer is a leading contributor to cancer incidence and mortality in women globally. Low- and middle-income countries (LMICs) experience a disproportionately higher burden of cervical cancer due to limited access to preventative and screening measures [1]. More than four out of every five new cases of cervical cancer in 2020 occurred in LMICs [2]. Persistent infection with high-risk human papillomavirus (HPV) types is the cause of nearly all cases of cervical cancer. Other HPV-associated cancers include cancer of the vagina, vulva, penis, anus, and oropharynx (back of throat) [3].

Vaccines to protect against HPV infection became available in 2006 and have been recommended by the World Health Organization (WHO) since 2009. They are also central to the WHO Global Strategy to Accelerate Cervical Cancer Elimination, which calls for 90 % of girls to be fully vaccinated against HPV by age 15 alongside screening and treatment targets to be achieved by the year 2030 [4]. Sustaining the cost of implementation is cited as a concern and cause of reluctance to introduce HPV vaccine in LMICs [5], [6]. This is particularly due to the fact that HPV vaccine delivery occurs predominately in school-based settings [7] outside of delivery platforms for infant vaccines, diminishing the possibility for economies of scope, when the same resources are spread across several interventions or antigens [8].

A prior systematic review published in 2022 focused on the programmatic costs and cost-effectiveness of HPV vaccination in all income settings [9], and another study published in 2019 examined delivery costs for routine immunizations—including HPV vaccines—in LMICs [10]. However, in both reviews, the included studies collected cost data during a program’s demonstration, pilot, or introduction phase and empirical evidence from national, routinized HPV vaccination programs was lacking. Recently, new data from immunization programs beyond introduction has become available, complementing these earlier cost estimates [12], [13]. Further, given recent global efforts to revitalize HPV vaccine delivery following the COVID-19 pandemic [14] and changes in the policy landscape due to the recommendation from WHO’s Strategic Advisory Group of Experts on Immunization that a single-dose HPV vaccination schedule may be considered for eligible populations [15], it is an appropriate time to furnish an updated review of HPV vaccine program costs by country, delivery strategy, and dosing schedule.

This systematic review summarizes the available evidence on the cost of HPV vaccine delivery in LMICs. Our synthesis aims to provide insights for country- and global-level policymakers on delivery costs at different stages of program maturity to inform budgeting and financing priorities for HPV vaccine introduction and program revitalization and sustainability.

## 2 Material and methods

### 2.1 Search strategy

We conducted a systematic review of published literature following PRISMA guidelines [16] to identify studies reporting the cost of HPV vaccine delivery in LMICs. Search terms included HPV, delivery, costing, and LMICs. We define LMICs as low-income, lower-middle-income, and upper-middle-income countries, as designated by the World Bank at the time the costing study took place [17]. We searched EconLit, Embase, Medline (via PubMed), Web of Science core collections, Google Scholar, WHO Global Index Medicus, and Immunization Delivery Cost Catalog for articles published in English between January 1, 2005, and November 10, 2023. This date range was selected as it spans the time when the first HPV vaccine demonstration projects occurred in LMICs. Search terms were adapted to each database as follows: EconLit, HPV AND cos* AND vaccin*; Embase, ('human papilloma virus vaccine'/exp OR 'human papilloma virus vaccine') AND 'delivery':ab AND ('cost analysis':ab OR 'cost evaluation':ab OR 'economic cos*':ab OR 'financial cos*':ab); Medline/PubMed, “HPV”[All Fields] AND “vaccin*”[All Fields] AND (“cost analysis”[All Fields] OR “microcos*”[All Fields] OR “economic cos*”[All Fields] OR “financial cos*”[All Fields]) AND (“delive*”[All Fields] OR “implemen*”[All Fields] OR “strateg*”[All Fields]); Web of Science core collections, (AB=(HPV Vaccin*) AND AB=(“cost analysis” OR “economic cos*” OR “financial cos*” OR “microcos*” OR “cost evaluation”) AND AB=(“implement*” OR “deliv*” OR “strateg*”)); Google Scholar, (“Cost analysis” AND (“HPV Vaccine delivery”)); WHO Global Index Medicus, HPV AND Vaccin* AND cos* AND (deliv* OR implement*).

We also searched for unpublished reports and gray literature using NGO Search and IGO Search [18], which are Google Custom Search tools developed by the American Library Association for this purpose. Search terms were as follows: “HPV vaccine” delivery “cost analysis”.

References of previous systematic reviews on this topic and of included articles were scanned for any additional relevant studies.

### 2.2 Eligibility criteria

We developed eligibility criteria using the Population, Intervention, Comparator, Outcome (PICO) framework [19], [20]. Studies were eligible for inclusion if the population was adolescents or preadolescents, the intervention was HPV vaccination, and the outcome was country-specific cost of HPV vaccine delivery. An explicit comparator was not required. Only studies of HPV vaccine delivery programs in countries classified as low income, lower-middle income, or upper-middle income at the time the study was conducted were included. Studies that did not report primary costing data or unit costs were excluded, as were studies for which full texts were not available in English. We defined primary costing data as data collected prospectively or retrospectively from a sample of immunization sites or administrative offices [21]; studies that report primary costing data may also include secondary costing inputs to supplement available primary data. PICO criteria are listed in Supplemental Table 1.

### 2.3 Selection process

Search results were uploaded to CADIMA [22], an open source systematic review software tool. This tool was selected because it is open-access and could be used to manage each stage of the review process, including linking all references identified to the search that generated them. References were deduplicated prior to screening, first through automatic detection of potential duplicates by CADIMA and then through manual review. Systematic reviews were excluded after their references were examined for any secondary resources. All abstracts were screened by one reviewer (EC or CP) with CADIMA randomly selecting 50 % for blind and independent screening by a second reviewer (RS). Exclusions and disagreements were discussed by MM, RS, CP, and EC and any ties broken by MM. Since results of gray literature searches were not easily uploadable to CADIMA, and because the review team anticipated a low number of included resources, title and abstract review for these references took place outside of CADIMA, with 50 % randomly selected for a blind and independent review by a second reviewer; included references were uploaded prior to full text review. Full text articles were blindly and independently reviewed by two reviewers (EC or CP, and RS), with any ties broken by MM.

### 2.4 Data extraction process

The study team developed a data extraction template, with cost categories adapted from the Immunization Delivery Cost Catalogue (IDCC) [23]. Data elements extracted included country and income level, delivery strategy and target population, sampling strategy, details of the costing methodology used and types of costs and activities costed, and results in terms of unit costs. If a study reported costing outcomes from more than one geographic area or delivery strategy, an entry was recorded for each geographic area and delivery strategy. We only extracted data from activities completed (e.g., demonstration project, routine delivery), and we did not include modeled or projected costs in this systematic review. We entered extracted data into a Microsoft Excel template and uploaded it to CADIMA. Uploading the filled template made the latest version of results available to all team members in a central location.

We extracted unit costs on financial and economic costs per dose and per fully immunized child (FIC), including and excluding the cost of HPV vaccine and supplies, as reported in the study. Data extraction was performed by EC and reviewed by MM and RS.

### 2.5 Currency conversion

All unit costs reported in the studies were converted to 2022 United States Dollars (USD). Costs were first converted to costing year local currency units (LCUs) using the exchange rate reported in the study. In cases where the study did not report an exchange rate, World Bank official rates were used [24]. For exchange rates not available from the World Bank, exchangerates.org.uk was used [25]. LCUs were then inflated to 2022 using country consumer price index (CPI) [26] and converted to USD using 2022 exchange rates [24], [25]. For studies in countries with unstable currencies, with cumulative inflation greater than 50 % between 2017 and 2022 (Ethiopia, Sri Lanka, Zambia, and Zimbabwe), or with missing cost year CPI (Peru and Uganda), study year USD were inflated to 2022 USD using US CPI [26]. Supplemental Fig. 1 provides a visual description of the currency conversion process.

### 2.6 Critical appraisal

Since there is not a single standard set of criteria for the critical appraisal of costing studies, we adapted criteria from the Consolidated Health Economic Reporting Standards (CHEERS) checklist [27], the Global Health Cost Consortium (GHCC) Reference Case [28], a recently published WHO-led consensus statement on vaccine delivery costing [29], and a proposed quality framework for costing studies [30]. We aimed for an efficient set of criteria with “yes” or “no”, “stated” or “not stated”, etc. responses; each element was either reported or not. In studies that included multiple components, such as a costing study and a model for projecting future costs or a cost-effectiveness analysis, we focused on the elements described for the costing study alone based on primary data collection and not projections. Critical appraisal was performed by EC and CP and reviewed by MM and RS. The full list of criteria with definitions is included in Supplemental Table 3.

## 3 Results

### 3.1 Articles included in the systematic review

**Fig. 1:**
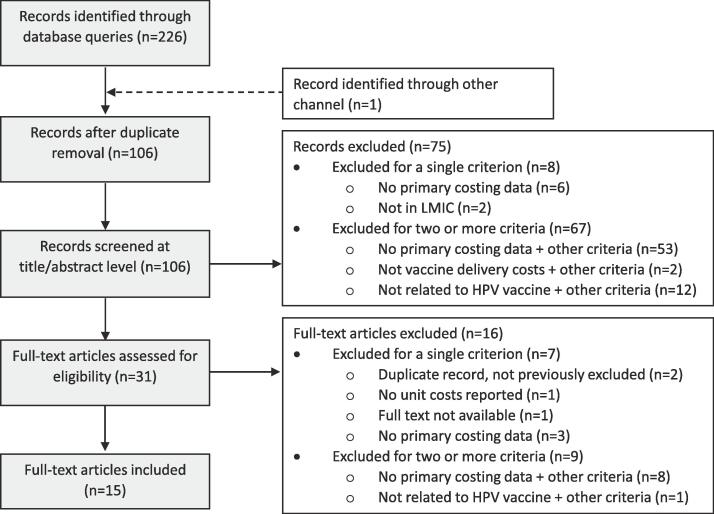
Flow diagram of included studies. Abbreviations: HPV = human papillomavirus, LMIC = Low- and middle-income countries.

Our literature search yielded 226 records and 1 record was identified from the references in one of the included studies. Following duplicate removal, 106 abstracts were screened, and 75 records were excluded, as described in Fig. 1. The remaining 31 full-text articles were reviewed and 2 required tie-breaking. A total of 16 full-text articles were excluded, resulting in a total of 15 studies included in our systematic review. These 15 studies included 13 peer-reviewed articles, 1 preprint, and 1 report*.*

### 3.2 Characteristics of the articles included in the review

**Fig. 2:**
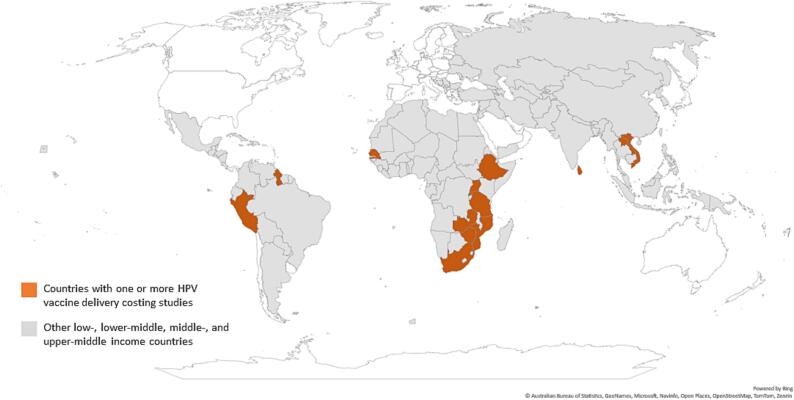
Geographic location of costing studies included in the systematic review. Abbreviation: HPV = human papillomavirus.

As shown in Fig. 2, 14 countries are represented in the studies reviewed, 64 % of which are in Africa. Key characteristics of the included studies are described in Table 1. The majority of studies are from the demonstration or pilot period (60 %) [31], [32], [33], [34], [35], [36], [37], [38], [39], costed a school-based delivery strategy (73 %) [11], [13], [31], [32], [33], [34], [35], [36], [37], [39], [40], and captured programs delivering a two-dose schedule (53 %) [11], [12], [13], [31], [34], [39], [41], [42], with the remainder costing a three-dose schedule. All studies represented HPV vaccination programs targeting girls aged 9 to 14 years (or equivalent grade level), and one study included a gender-neutral program [13]. All included studies assessed financial or economic costs, used ingredients-based microcosting approaches, and represented a similar perspective (i.e., payer, provider, health system, government, Ministry of Health, or project). Sample size varied by study. Sample size for health facilities ranged from 2 to 107, when stated. Some studies also included data collection from administrative levels such as districts, regions, and the national level. Other studies only stated the number of schools included in the study or the number of districts, with no details on the affiliated health facilities where vaccinators emanated from. Three studies did not state their sample size, with one study reporting that a staff member from each facility was interviewed about personnel costs [38] and the other two stating that stakeholder interviews took place at different levels of the health system [12], [33].

**Table 1:** Key characteristics of included costing studies.

First author (year)	Income group*	Country	Phase	Delivery strategy	Schedule evaluated	Target population for vaccination	Study reference period	Timing of costing	Costing type		Costs included		Ingredients-based	Perspective	Sample size
									Full	Inc.	Fin.	Econ.			
Alonso et al. (2019)	LIC	Mozambique	Demo	School-based	3 doses	10-year-old girls	2014	Prospective (concurrent)	X	X	X	X	Yes	Health system	Not stated; conducted key informant interviews at national, district, and health facility levels
Soi et al. (2019)	LIC	Mozambique	Demo	School-based	2 doses (3 doses in year 1)	10-year-old girls	2014–2017	Prospective (concurrent)		X†	X†	X†	Yes†	Payer	40 health facilities and 3 districts
Levin et al. (2013)	LMICs	Peru, Uganda, and Vietnam	Demo	School-based with facility-based and outreach, varying by country	3 doses	10- to 11-year-old girls and Grade 5 and 6 girls, varying by country	2008–2009, varying by country	Retrospective†		X	X	X	Yes	Government	12–14 health facilities, varying by country
Moodley et al. (2016)	UMIC	South Africa	Demo	School-based	3 doses	Girls in grade 4	2014†	Retrospective		X	X	X	Yes	Health system†	1 district
Van Minh et al. (2017)	LMIC	Vietnam	Demo	Facility-based†	3 doses	11-year-old girls	Not stated	Prospective†		X	X		Yes	Provider	Not stated
Hidle et al. (2018)	LMIC	Zimbabwe	Demo	School-based	2 doses	10-year-old girls	2014–2016	Retrospective		X	X	X	Yes	Provider	2 districts
Asfaw (2017)	LIC	Ethiopia	Pilot	School-based	2 doses	Girls in grade 4 and 10-year-old girls out of school	2015–2016	Retrospective†		X	X	X	Yes	Government	4 health facilities, 2 districts, 2 regions, and 1 national level with various interviews at each level
Riewpaiboon et al. (2019)	LMIC	Lao PDR	Pilot	School-based	3 doses	Girls in grade 5 and 10-year-old girls out of school	2013–2014	Prospective (concurrent)		X	X	X	Yes	Ministry of Health	107 health facilities, 22 districts, and 2 provinces
Quentin et al. (2012)	LIC	Tanzania	Pilot	School-based	3 doses	Class 6 girls or girls born in 1998	2010–2011	Prospective (concurrent)		X	X	X	Yes	Project	134 schools
Ngabo et al. (2015)	LIC	Rwanda	Intro	School-based	3 doses	Girls in P6 and 12-year-old girls out of school	2012	Retrospective		X	X	X	Yes	Government	2 health facilities
Simuyemba et al. (2023)	LMIC	Zambia	Intro	Mixed	2 doses†	14-year-old girls	2019–2020†	Retrospective†		X†	X	X	Yes (with some top-down costing)	Ministry of Health†	8 districts, 4 provinces, and 1 national level
Brennan et al. (2022)	LMIC	Senegal	Intro and routine	Mixed	2 doses	9-year-old girls	2018–2020	Retrospective		X	X	X	Yes	Provider	77 health facilities, 31 districts, and 14 regions
Hidle et al. (2022)	LMIC	Zimbabwe	Intro and routine	School-based	2 doses	10-to 14-year-old girls	2018–2019	Retrospective		X	X	X	Yes	Provider	60 health facilities and 30 districts
Mvundura et al. (2023)	LMICs	Ethiopia, Guyana, Rwanda, Senegal, Sri Lanka, and Uganda	Routine	School-based and mixed, varying by country	2 doses	9-to 14-year-old girls, varying by country, and boys included in Guyana	2019	Retrospective		X	X	X	Yes	Health system	30–66 health facilities per country, varying by country; 5–29 subnational administrative offices per country, varying by country; and 1 national office per country
Hsiao et al. (2023)	LMIC	Tanzania	Routine	Mixed	2 doses	14-year-old girls	2018–2022†	Retrospective		X	X	X	Yes	Government	Not stated; conducted key informant interviews at regional, district, and health facility levels

Abbreviations: demo = demonstration, econ. = economic, finan. = financial, inc. = incremental, intro = introduction, LIC = low-income country, LMIC = low- and middle-income country, UMIC = upper middle-income country.

*At the time of the costing study reference period.

†Inferred by systematic reviewers.

The type of costs and activities included in the studies varied across studies, with all studies including transportation and fuel and health worker time as cost types and training and social mobilization and IEC as activities, as shown in Supplemental Table 4. The least included inputs were the capital cost of vehicles and non-health worker time (e.g., volunteers and school staff). Regarding the classification of costs as financial or opportunity costs, differences in the payer and perspective of the evaluation impacted the classification. When reporting outputs, of the studies in our sample, five reported costs by activity only [31], [32], [33], [34], [40], two by cost type only [35], [42], seven reported costs by both activity and cost category [11], [12], [13], [37], [39], [41], [42], and one did not report their results in detail [38].

### 3.3 Estimated financial and economic costs in 2022 US$

**Fig. 3:**
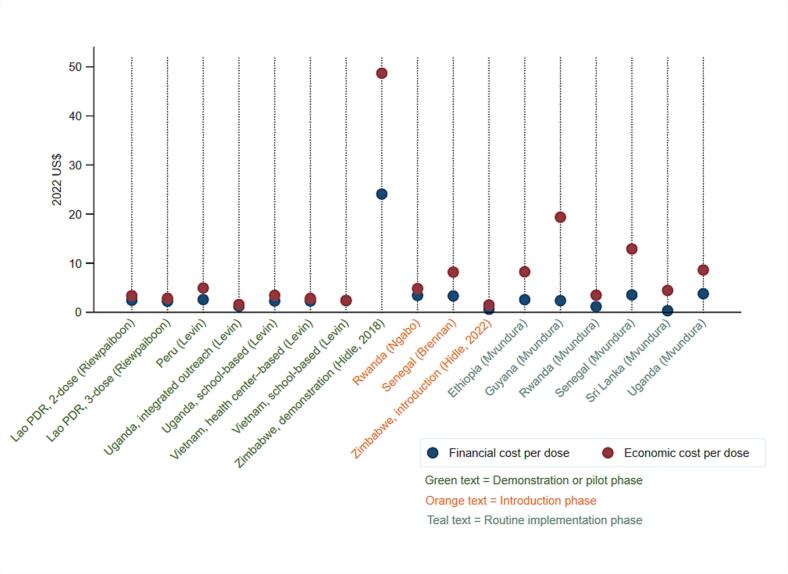
Financial and economic costs per dose for HPV vaccine delivery, excluding vaccine supplies and procurement costs, in 2022 US$.

Extracted cost data are shown in Table 2. Economic costs per FIC, including vaccine and injection supplies procurement costs, and financial costs per dose, excluding vaccine and injection supplies procurement costs, were the most frequently reported outcomes. These unit costs were each reported by 8 of the 15 studies. Fig. 3 displays the adjusted delivery costs per dose in 2022 US$, excluding vaccine procurement costs, and ordered by phase of implementation (i.e., demonstration, pilot, introduction, and routine). The lowest financial cost per dose of $0.31 is reported for Sri Lanka’s routine school-based HPV vaccination program [13], while the highest financial cost per dose of $24.70 is reported for Zimbabwe’s school-based demonstration program [39]. The Zimbabwe demonstration program also reports the highest economic cost per dose at $48.70 [39], although the introduction and routine vaccination program in Zimbabwe reports the lowest economic cost per dose at $1.48 [11]. The majority of the studies evaluated costs for school-based delivery, and there is limited evidence on costs for delivery in non-school-based community settings or health facilities.

**Table 2:** Extracted unit cost estimates from included studies, in US$ in stated currency year.

First author (year)	Country	Study subgroup, if reported	Currency year for costs	Cost estimates (including vaccine cost) if reported				Cost estimates (excluding vaccine cost) if reported			
				Financial cost per dose	Economic cost per dose	Financial cost per FIC	Economic cost per FIC	Financial cost per dose	Economic cost per dose	Financial cost per FIC	Economic cost per FIC
Alonso et al. (2019)	Mozambique	Full study	2014		$17.59		$52.29	$6.07		$17.95	
Soi et al. (2019)	Mozambique	Full study	2014				$54.00				
		Full study (2-dose schedule only)	2014				$48.00				
		Manhiça district	2014				$42.00				
		Manica district	2014				$49.00				
		Mocimboa da Praia district	2014				$222.00				
Levin et al. (2013)	Peru, Uganda, and Vietnam	Peru school-based	2009					$2.03	$3.88		
		Uganda school-based	2009					$2.10	$3.15		
		Uganda integrated outreach	2009					$1.11	$1.44		
		Vietnam school-based	2009					$1.62	$2.08		
		Vietnam health center-based	2009					$1.55	$1.92		
Moodley et al. (2016)	South Africa	Full study	2014			$46.39					
Van Minh et al. (2017)	Vietnam	Full study	2012*							$10.40	
Hidle et al. (2018)	Zimbabwe	Full study	2016	$19.76	$45.00	$40.03	$91.19	$19.74	$39.94	$40.00	$80.93
Asfaw (2017)	Ethiopia	Ahefrom district	2016*			$13.30	$33.40			$11.71	$21.82
		Gomma district	2016*			$6.68	$20.48			$5.12	$9.19
Riewpaiboon et al. (2019)	Lao PDR	3-dose schedule	2013					$2.62	$3.33	$7.87	$9.99
		2-dose schedule	2013					$2.85	$3.95	$5.70	$7.92
Quentin et al. (2012)	Tanzania	Urban class-based	2011				$66.00				
		Urban age-based	2011				$100.00				
		Rural class-based	2011				$78.00				
		Rural age-based	2011				$107.00				
Ngabo et al. (2015)	Rwanda	Full study	2012		$11.73	$11.93	$35.66	$3.37	$4.76	$10.23	$14.45
Simuyemba et al. (2023)	Zambia	Full study	2020	$6.00	$23.00	$11.90	$46.00				
		Health facility-based	2020	$94.70	$365.20	$189.40	$730.40				
		Outreach	2020	$30.80	$118.80	$61.60	$237.70				
		Schools	2020	$3.40	$13.20	$6.80	$26.40				
Brennan et al. (2022)	Senegal	Full study	2020	$7.75	$12.24			$3.07	$7.56		
Hidle et al. (2022)	Zimbabwe	Full study	2020					$0.53	$1.31		
Mvundura et al. (2023)	Ethiopia, Guyana, Rwanda, Senegal, Sri Lanka, and Uganda	Ethiopia	2019					$2.23	$7.19		
		Guyana	2019					$2.10	$17.20		
		Rwanda	2019					$1.03	$3.09		
		Senegal	2019					$3.27	$11.94		
		Sri Lanka	2019					$0.27	$3.88		
		Uganda	2019					$3.32	$7.58		
Hsiao et al. (2023)	Tanzania	Full study	2019	$2.22	$10.01	$5.17	$23.34				

Abbreviations: FIC = fully immunized child.

*Inferred by systematic reviewers.

### 3.4 Costs by HPV vaccination program implementation phase

When we examine the delivery costs per dose adjusted to 2022 US$, excluding vaccine procurement costs, there is no clear pattern on how costs change by implementation phase. Considering financial cost per dose, the range is $1.21 to $24.07 for pilot or demonstration (n = 4 studies), $0.60 to $3.41 for introduction (n = 3 studies), and $0.31 to $3.77 for routine implementation (n = 1 study) as shown in Supplemental Table 5. The ranges are similarly overlapping for economic costs per dose, with $1.57 to $48.70 for pilot or demonstration, $1.48 to $8.17 for introduction, and $3.47 to $19.36 for routine implementation.

In Rwanda, Senegal, Uganda, and Zimbabwe, multiple studies were conducted over different phases of implementation. In Rwanda and Zimbabwe, cost estimates decreased between demonstration or introduction, with financial costs per dose in Zimbabwe estimated at $24 during demonstration [39] and $0.60 during introduction [11]. Conversely, cost per dose estimates modestly increased with program maturity in Senegal [13], [41] and Uganda [13], [35].

### 3.5 Evidence of variations in costs by geography or delivery setting within the same country

Four articles provide data by different geography or delivery strategy within the same country, as shown in Table 2. A study of a pilot program in Ethiopia found that financial cost per FIC in one district was nearly double that in another [31]. In Mozambique, economic cost per FIC, including HPV vaccine procurement costs, in one district were more than double the same unit cost in the other three districts [34]. In both cases, the district(s) with the larger target population size or greater number of doses delivered had the lower costs per dose [31], [34]. Two articles report costs disaggregated by delivery strategy [35], [42]. In Zambia, financial and economic costs per dose were lowest for HPV vaccine delivery in schools where a greater number of doses were delivered [42]. In Uganda, integrated outreach delivery reported lower costs per dose when compared to school-based delivery, and in Vietnam, HPV vaccine delivery cost in health centers was lower than delivery cost in schools [35]. In these two countries, the delivery strategy with the lowest annual delivery costs reported the lowest cost per dose [35].

### 3.6 Cost types and activities contributing a large share of the costs

For studies reporting findings by cost types and that exclude the cost of HPV vaccine, human resource time represents the largest share of reported economic costs, or the largest share of recurrent costs [11], [13], [35], [41]. In two studies that did include vaccine costs, personnel time still accounted for the largest single contributor to economic costs [37], [42]. The majority of studies that include vaccine costs report that vaccine procurement (activity) or the cost of vaccines and injection supplies (cost type) contributed most significantly to financial or economic costs [12], [33], [34], [36], [39], [40]. The activity of service delivery also contributed a large share of the financial and or economic costs in studies reporting costs by activity [11], [12], [13], [32], [33], [39], [40], [41].

### 3.7 Findings from the quality assessment

Results of the quality assessment are presented in Table 3. All of the studies in this systematic review reported on the study purpose/objective, audience, target population, cost estimates, phase of implementation when costing was done, and specified the type of costs included. Just over half of the studies described the sampling method for costing. Similarly, key data such as the study’s time horizon (or reference period), data sources for prices, and whether the study was prospective or retrospective were not stated in 27 % of the studies. The uncertainty of reported delivery cost estimates and whether HPV vaccine delivery was stand-alone or jointly with other vaccines/interventions were the lowest reported criteria we assessed, with only 40 % of studies reporting on these.

**Table 3:** Results of critical appraisal.

First author (year)	Purpose/objective stated	Audience stated	Time horizon stated	Types of costs included are specified/defined	If pilot or demonstration program, startup vs non-startup costs defined	If incremental costing, assumptions about health system capacity described	Scope of inputs estimated defined, including boundaries/exclusion criteria	Method for measuring/estimating each input defined	Data source used to measure units described	Data source used for prices stated	Depreciation approach stated	Currency, including any conversions or inflation, reported	Variation in costs and drivers of variation reported	Uncertainty of estimates characterized	Cost estimates communicated	Delivery strategy stated	Phase when costing was done stated	Standalone vs joint delivery stated	Sampling method described	Perspective stated	Prospective vs retrospective stated	Full vs incremental costing stated	Financial costs	Economic costs	Target population defined
Alonso et al. (2019)	S	S	S	Yes	D	D	Yes	Yes	Yes	Yes	Yes	Yes	Yes	No	Yes	S	S	S	ND	S	S	S	Included	Included	D
Asfaw (2017)	S	S	S	Yes	ND	D	Yes	Yes	No	No	No	No	Yes	No	Yes	S	S	NS	ND	S	NS	S	Included	Included	D
Brennan et al. (2022)	S	S	S	Yes	N/A	D	Yes	Yes	Yes	Yes	Yes	Yes	No	Yes	Yes	S	S	NS	D	S	S	S	Included	Included	D
Hidle et al. (2018)	S	S	S	Yes	D	D	Yes	Yes	Yes	Yes	No	Yes	Yes	No	Yes	S	S	S	D	S	S	S	Included	Included	D
Hidle et al. (2022)	S	S	S	Yes	N/A	D	Yes	Yes	Yes	Yes	Yes	Yes	Yes	Yes	Yes	S	S	NS	D	S	S	S	Included	Included	D
Hsiao et al. (2023)	S	S	NS	Yes	N/A	D	Yes	Yes	Yes	Yes	Yes	Yes	Yes	Yes	Yes	S	S	NS	ND	S	S	S	Included	Included	D
Levin et al. (2013)	S	S	S	Yes	D	D	Yes	Yes	Yes	No	Yes	Yes	Yes	No	Yes	S	S	NS	D	S	NS	S	Included	Included	D
Moodley et al. (2016)	S	S	NS	Yes	ND	ND	Yes	No	No	No	Yes	Yes	Yes	Yes	Yes	S	S	S	ND	NS	S	S	Included	Included	D
Mvundura et al. (2023)	S	S	S	Yes	N/A	D	Yes	Yes	Yes	Yes	Yes	Yes	Yes	Yes	Yes	S	S	NS	D	S	S	S	Included	Included	D
Ngabo et al. (2015)	S	S	S	Yes	D	D	Yes	Yes	Yes	Yes	Yes	Yes	Yes	No	Yes	S	S	S	ND	S	S	S	Included	Included	D
Quentin et al. (2012)	S	S	S	Yes	D	ND	Yes	Yes	Yes	Yes	Yes	Yes	Yes	No	Yes	S	S	S	D	S	S	S	Included	Included	D
Riewpaiboon et al. (2019)	S	S	S	Yes	D	D	Yes	Yes	Yes	Yes	Yes	Yes	Yes	No	Yes	S	S	NS	D	S	S	S	Included	Included	D
Simuyemba et al. (2023)	S	S	NS	Yes	N/A	ND	Yes	Yes	Yes	Yes	No	Yes	Yes	No	Yes	S	S	NS	ND	NS	NS	S	Included	Included	D
Soi et al. (2019)	S	S	S	Yes	D	D	Yes	Yes	Yes	Yes	No	Yes	Yes	Yes	Yes	S	S	NS	D	S	S	NS	Not included	Not included	D
Van Minh et al. (2017)	S	S	NS	Yes	D	ND	Yes	No	No	No	Yes	No	No	No	Yes	NS	S	S	ND	S	NS	NS	Included	Not included	D

Abbreviations: S = stated, NS = not stated, D = defined or described, ND = not defined or not described.

## 4 Discussion

This review provides a summary of the current evidence on HPV vaccine delivery costs, from 15 studies representing 14 countries. This includes costs from two studies that represent seven countries past the introduction years. Financial cost per dose, excluding vaccine and supplies, ranged from $0.31 to $24.07 per dose, while economic cost per dose ranged from $1.48 to $48.70 per dose in 2022 US$.

When we consider study characteristics, the majority of studies in our review costed HPV vaccine delivery during the demonstration, pilot, or introduction phase; costed delivery of a three- or two-dose schedule; and took place in countries in Africa. There is no empirical evidence on HPV vaccine delivery using a single-dose schedule, but modeling suggests an increase in the cost per dose—given that total costs (numerator) would decrease less substantially than the number of doses delivered (denominator)—but a lower cost per FIC [12], [43]. Future research on HPV vaccine delivery of a single-dose schedule would supplement modeled estimates and provide evidence on the magnitude of the change in costs based on actual implementation. Similarly, more studies from outside of Africa would be a welcome addition to the literature. There is limited to no evidence on HPV vaccine delivery costs from LMICs in Europe, the Middle East, and Latin America and the Caribbean regions.

Based on our review, there is no clear pattern with delivery costs and phase of implementation. While in some countries, such as Rwanda and Zimbabwe, HPV vaccine delivery costs per dose decline with time since introduction, the opposite is true in Uganda and Senegal. In Uganda, differences in estimates were due to coverage improvement activities conducted in the country during the year of the routine costing study, and in Senegal, a catch-up campaign during introduction led to more doses delivered during the introduction phase, contributing to the variation in cost per dose estimates [13]. It is possible that differences in study methods could also explain some of these differences in research findings. Additional research, particularly costing studies using the same methodology in the same country over time, would provide improved evidence on the impact of program maturity on costs and how costs change over time.

Only six of the studies in our review reported a sample size of 30 or greater at the health facility level [11], [13], [32], [34], [37], [41], which impacts the robustness of the cost estimates generated and may mask within-country variability. In the few studies that reported delivery costs by district, we see substantial differences in costs across the geographies reported. Future research on HPV vaccine delivery costs should consider and report in-country variability in cost estimates, as these findings are relevant for planning and financing HPV vaccination programs.

As previously noted, the studies in our review estimated delivery costs for predominantly school-based strategies and two studies provided unit costs disaggregated by delivery strategy in the same country. However, the studies do not report on the relationship between costs and coverage and the cost of strategies to reach out-of-school children. As countries work to revitalize HPV vaccination programs following the COVID-19 pandemic, costing studies of strategies to increase coverage will be helpful to guide decision-making.

Our findings are generally comparable to prior systematic reviews, with some differences in inclusion criteria, data extracted, and additions to the evidence base. All five articles reported by Akumbom et al. [9] are similarly reported in our review. We also present overlapping results with the systematic review carried out by Vaughan et al. [10], including five of the seven studies they cite related to HPV vaccine delivery. The two articles we did not report were excluded from our review, as they did not include primary costing data, which was one of our inclusion criteria. Lastly, we have included recent publications not previously included in the prior systematic reviews, thus updating the evidence base [12], [13], [41].

When considering the quality of reporting, we find that the majority of studies in our review did not adequately describe or justify sampling methods nor sufficiently report uncertainty of the costing estimates, such as including confidence intervals. These same issues were also identified in the WHO-led consensus statement on vaccine delivery costing, and reporting adequately on these points is included in their recommendations [29]. Further, they recommend defining vaccine delivery costs as exclusive of vaccine procurement costs [29] however, one third of the studies in our review only report cost estimates inclusive of vaccine procurement costs. As discussed in prior research, methodological differences in costing studies—including the precise scope of inputs—continue to make direct comparisons challenging [29], [30]. We found that studies may specify cost types or activities evaluated in their studies, but not report findings disaggregated by these same measures, nor report whether a particular cost type is considered a financial, opportunity, or economic cost, further complicating results comparison across studies. Comparability would be enhanced if studies clearly defined which costs inputs were financial, opportunity, and economic, and reported outputs by the same activities and cost types defined in the methods. Further, multi-country studies that apply the same sampling, data collection, analysis methods, and reporting rubric across settings would facilitate more direct comparison. Only two studies in our review were carried out in multiple countries [13], [35]; future research including a variety of geographies would be a welcome addition to the literature.

Our systematic review has limitations. Based on our inclusion criteria, we only considered studies for which the full-text publication was available in English, limiting inclusion of research available in other languages. Second, our systematic review focused on primary cost data for HPV vaccine delivery activities conducted; additional cost estimates from modeling studies or costing tool projections are present in the literature but were outside the scope of this review. Third, we did not do blind independent review of all abstracts under consideration, which introduced a chance of error or omission in the final included studies. However, given the specificity and objectivity of our criteria, we believe the chance of error is low and our findings capture the available published literature on HPV vaccines delivery costs, as defined by our inclusion criteria. In the process of carrying out the critical appraisal, we observed that some studies provided well-detailed methodology with careful mapping of the scope of inputs included, for example, while other papers provided only a brief narrative overview of this criterion. Both of these studies, however, would elicit a “yes” for the critical appraisal, elucidating a weakness of the checklist-based criteria.

## 5 Conclusions

This systematic review summarizes the available evidence based on primary data collection on the cost of HPV vaccine delivery in LMICs. All currently available cost estimates are based on a two- or three-dose vaccination schedule. We found that delivery costs vary across and within countries and by phase of implementation. Our review also highlights areas for further research including HPV vaccine delivery studies in more countries outside of Africa, as well as costing studies for a single-dose schedule and of strategies to improve coverage.

## Funding

This work was also supported, in whole or in part, by the Bill & Melinda Gates Foundation [INV-005053]. Under the grant conditions of the Foundation, a Creative Commons Attribution 4.0 Generic License has already been assigned to the Author Accepted Manuscript version that might arise from this submission.

## CRediT authorship contribution statement

**Rose Slavkovsky:** Formal analysis, Methodology, Validation, Writing – original draft, Writing – review & editing. **Emily Callen:** Formal analysis, Investigation, Methodology, Writing – original draft, Writing – review & editing. **Clint Pecenka:** Conceptualization, Formal analysis, Funding acquisition, Project administration, Validation, Writing – review & editing. **Mercy Mvundura:** Conceptualization, Funding acquisition, Methodology, Supervision, Validation, Writing – review & editing.

## Declaration of competing interest

The authors declare the following financial interests/personal relationships which may be considered as potential competing interests: Mercy Mvundura and Rose Slavkovsky were authors on one paper (Mvundura et al.) included in the systematic review; MM and RS did not perform the critical appraisal for their own work. If there are other authors, they declare that they have no known competing financial interests or personal relationships that could have appeared to influence the work reported in this paper.

## Data Availability

All data reported is available in the manuscript tables including supplementary materials.
