# Supplementary material for: Costs of human papillomavirus vaccine delivery in low- and middle-income countries: A systematic review
Source: Vaccine. 2024 Feb 27;42(6):1200–10. doi: 10.1016/j.vaccine.2024.01.094 (PMC10911079; doi:10.1016/j.vaccine.2024.01.094)
Supplement: Supplementary Data 1 [file mmc1.docx]

**Supplementary appendix**

Table 1. PICO framework for inclusion criteria, used during abstract and full-text review in CADIMA.

| **Criteria** | **Key element** |
| --- | --- |
| Population | Adolescents |
| Population | Low income, lower-middle income, or upper-middle income country |
| Intervention | HPV vaccination |
| Comparator | Not required |
| Outcome | Primary costing data |
| Outcome | Country-specific |
| Outcome | Vaccine delivery cost (unit costs reported) |

Abbreviation: PICO = Population, Intervention, Comparator, and Outcome

Figure 1. Currency conversion methods used.


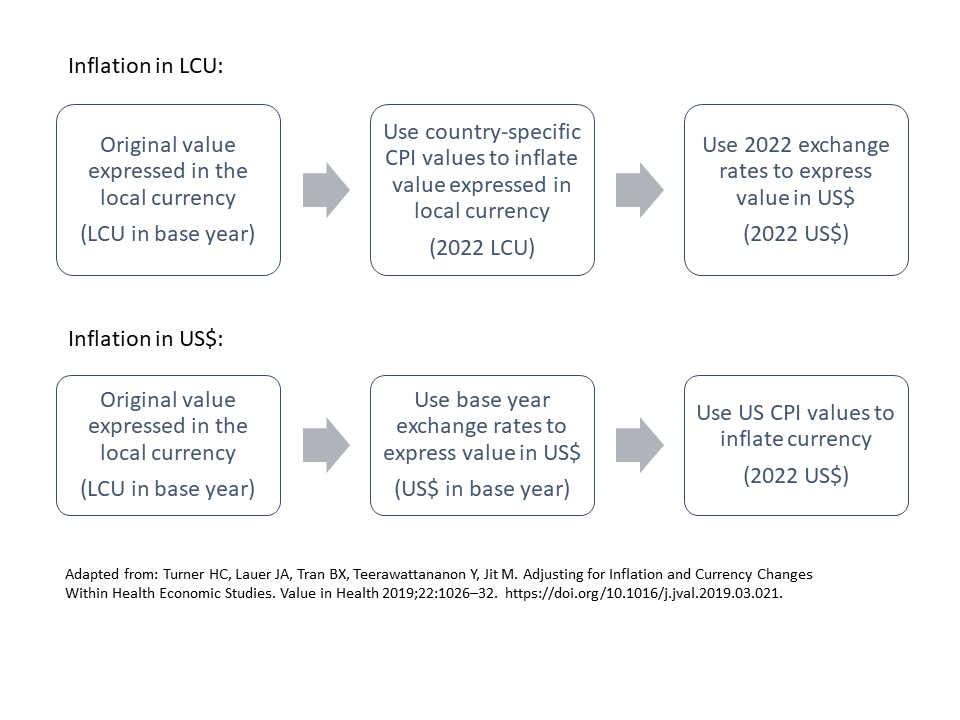


Abbreviations: LCU = local currency unit

Table 2. Reference list of included studies.

| **#** | **Reference** | **Countries** | **# unit costs extracted** |
| --- | --- | --- | --- |
| 1 | Alonso S, Cambaco O, Maússe Y, Matsinhe G, Macete E, Menéndez C, et al. Costs associated with delivering HPV vaccination in the context of the first year demonstration programme in southern Mozambique. BMC Public Health 2019;19. https://doi.org/10.1186/s12889-019-7338-4. | Mozambique | 4 |
| 2 | Asfaw E. Costing the Human Papillomavirus Vaccine Introduction in Ethiopia. Unpublished report. 2017. | Ethiopia | 4 |
| 3 | Brennan T, Hidle A, Doshi RH, An Q, Loharikar A, Casey R, et al. Cost of human papillomavirus vaccine delivery in a single-age cohort, routine-based vaccination program in Senegal. Vaccine 2022;40:A77–84. https://doi.org/10.1016/j.vaccine.2021.11.057. | Senegal | 4 |
| 4 | Hidle A, Gwati G, Abimbola T, Pallas SW, Hyde T, Petu A, et al. Cost of a human papillomavirus vaccination project, Zimbabwe. Bull World Health Organ 2018;96:834–42. | Zimbabwe | 8 |
| 5 | Hidle A, Brennan T, Garon J, An Q, Loharikar A, Marembo J, et al. Cost of human papillomavirus vaccine delivery at district and health facility levels in Zimbabwe: A school-based vaccination program targeting multiple cohorts. Vaccine 2022;40:A67–76. | Zimbabwe | 2 |
| 6 | Hsiao A, Struckmann V, Stephani V, Mmbando D, Changalucha J, Baisley K, et al. Costs of delivering human papillomavirus vaccination using a one- or two-dose strategy in Tanzania. VACCINE 2023;41:372–9. https://doi.org/10.1016/j.vaccine.2022.11.032. | Tanzania | 4 |
| 7 | Levin C, Minh H, Odaga J, Rout S, Ngoc D, Menezes L, et al. Delivery cost of human papillomavirus vaccination of young adolescent girls in Peru, Uganda and Viet Nam. Bull WORLD Health Organ 2013;91:585–92. https://doi.org/10.2471/BLT.12.113837. | Peru, Uganda, Vietnam | 10 |
| 8 | Moodley I, Tathiah N, Sartorius B. The costs of delivering human papillomavirus vaccination to Grade 4 learners in KwaZulu-Natal, South Africa. S Afr Med J 2016;106:497. https://doi.org/10.7196/SAMJ.2016.v106i5.9988. | South Africa | 1 |
| 9 | Mvundura M, Slavkovsky R, Debellut F, Naddumba T, Bayeh A, Ndiaye C, et al. Cost and operational context for national human papillomavirus (HPV) vaccine delivery in six low- and middle-income countries. Vaccine 2023;41:7435–43. https://doi.org/10.1016/j.vaccine.2023.11.008. | Ethiopia, Guyana, Rwanda, Senegal, Sri Lanka, Uganda | 12 |
| 10 | Ngabo F, Levin A, Wang SA, Gatera M, Rugambwa C, Kayonga C, et al. A cost comparison of introducing and delivering pneumococcal, rotavirus and human papillomavirus vaccines in Rwanda. Vaccine 2015;33:7357–63. https://doi.org/10.1016/j.vaccine.2015.10.022. | Rwanda | 7 |
| 11 | Quentin W, Terris-Prestholt F, Changalucha J, Soteli S, Edmunds WJ, Hutubessy R, et al. Costs of delivering human papillomavirus vaccination to schoolgirls in Mwanza Region, Tanzania. BMC Med 2012;10. https://doi.org/10.1186/1741-7015-10-137. | Tanzania | 4 |
| 12 | Riewpaiboon A, Pathammavong C, Fox K, Hutubessy R. Cost analysis of pilot school-based HPV vaccination program in two provinces of Lao PDR. Pharm Sci Asia 2019;46:46–53. https://doi.org/10.29090/psa.2019.01.017.0052. | Lao PDR | 8 |
| 13 | Simuyemba MC, Chama-Chiliba CM, Chompola A, Sinyangwe A, Bchir A, Asiimwe G, et al. An Evaluation of the Cost of Human Papilloma Virus (HPV) Vaccine Delivery In Zambia. In Review; 2023. https://doi.org/10.21203/rs.3.rs-2919637/v1. | Zambia | 16 |
| 14 | Soi C, Babigumira JB, Chilundo B, Muchanga V, Matsinhe L, Gimbel S, et al. Implementation strategy and cost of Mozambique’s HPV vaccine demonstration project. BMC Public Health 2019;19. https://doi.org/10.1186/s12889-019-7793-y. | Mozambique | 5 |
| 15 | Van Minh H, My NTT, Jit M. Cervical cancer treatment costs and cost-effectiveness analysis of human papillomavirus vaccination in Vietnam: a PRIME modeling study. BMC Health Serv Res 2017;17:353. https://doi.org/10.1186/s12913-017-2297-x. | Vietnam | 1 |

Table 3. Critical appraisal criteria, source, and definition.

| **#** | **Item** | **Source(s)** | **Definition of the criteria** |
| --- | --- | --- | --- |
| 1 | Purpose/objective | GHCC [1], CHEERS [2], Vaughan et al. [3], WHO [4] | From GHCC: “Why the analysis is being done, which may include financial planning, budgeting, policy decision making, etc.” |
| 2 | Audience | GHCC [1], CHEERS [2], Vaughan et al. [3], WHO [4] | Intended users or consumers of the analysis; may be implicit or explicit |
| 3 | Time horizon | GHCC [1], CHEERS [2], Vaughan et al. [3], WHO [4] | From GHCC: “The length of time of service provision or implementation that the costs are being considered” |
| 4 | Types of costs included are specified/defined | WHO [4] | States whether economic vs. financial costs and incremental vs. full costs are included |
| 5 | If pilot or demonstration, startup vs. non-startup costs defined | GHCC [1], Vaughan et al. [3], WHO [4] | Which costs were classified as startup should be defined, not just whether startup costs were included |
| 6 | If incremental costing, assumptions about health system capacity described | GHCC [1] | For example, will existing cold chain or transport infrastructure be used |
| 7 | Scope of inputs estimated defined, including and boundaries/exclusion criteria | WHO [4], GHCC [1] | Includes a description of how inputs were determined or mapped, and any exclusions (which could include costs at different/higher administrative levels) |
| 8 | Method for measuring/estimating each input defined | GHCC [1], CHEERS [2], Vaughan et al. [3], WHO [4] | Allocation method used for each input given (whether bottom up or top down), how human resources are measured |
| 9 | Data source used to measure units described | GHCC [1], Vaughan et al. [3], WHO [4] | Sources of units of resource use costed are reported, which may be aggregated |
| 10 | Data source used for prices stated | GHCC [1], CHEERS [2], Vaughan et al. [3], WHO [4] | Sources of prices are reported, including salaries and wages |
| 11 | Depreciation approach stated | GHCC [1], Vaughan et al. [3], WHO [4] | If capital inputs were included, how they were depreciated over the study timeframe |
| 12 | Currency, including any conversions or inflation, reported | GHCC [1], CHEERS [2], Vaughan et al. [3], WHO [4] | Any price adjustments are transparent; currency year should be reported |
| 13 | Variation in costs and drivers of variation reported | GHCC [1], WHO [4] | Can include differences across sites, target populations, strategies, etc. |
| 14 | Uncertainty of estimates characterized | GHCC [1], CHEERS [2], Vaughan et al. [3], WHO [4] | From GHCC: “For studies with multiple sites, should at minimum include an assessment of precision (confidence intervals or percentiles)” |
| 15 | Cost estimates communicated | GHCC [1], CHEERS [2], Vaughan et al. [3], WHO [4] | Total costs and/or unit costs are clearly reported |
| 16 | Delivery strategy | Vaughan et al. [3] | How the vaccine was delivered (school-based, facility-based, outreach, or something else) should be described |
| 17 | Phase when costing was done stated | GHCC [1], Vaughan et al. [3], WHO [4] | Important for comparing across studies: was vaccine delivery in pilot, demonstration, or routine delivery phase |
| 18 | Standalone vs. joint delivery | GHCC [1] | Was the vaccine delivered in conjunction with another program, or as a separate project |
| 19 | Sampling method described | GHCC [1], Vaughan et al. [3], WHO [4] | How sites were chosen stated |
| 20 | Perspective | GHCC [1], CHEERS [2], Vaughan et al. [3], WHO [4] | Generally societal or provider, but should include the groups or payers whose cost is captured |
| 21 | Retrospective vs. prospective stated | GHCC [1], CHEERS [2], Vaughan et al. [3], WHO [4] | Timing of data collection relative to timing of program implementation |
| 22 | Full vs. incremental costing described | GHCC [1], Vaughan et al. [3], WHO [4] | Component of scope, is the costing approach clear |
| 23 | Financial costs included | GHCC [1], Vaughan et al. [3], WHO [4] | Whether these costs were clearly included |
| 24 | Economic costs included | GHCC [1], Vaughan et al. [3], WHO [4] | Whether these costs were clearly included |
| 25 | Target population | GHCC [1], CHEERS [2], Vaughan et al. [3], WHO [4] | Whether the target population was clearly defined, may or may not include size of the target population |

Abbreviations: CHEERS = Consolidated Health Economic Evaluation Reporting Standards, GHCC = Global Health Cost Consortium, WHO = World Health Organization

References:

[1] Vassall A, Sweeney S, Kahn JG, Gomez G, Bollinger L, Marseille E, et al. Reference Case for Estimating the Costs of Global Health Services and Interventions n.d.

[2] Husereau D, Drummond M, Augustovski F, Bekker-Grob E de, Briggs AH, Carswell C, et al. Consolidated Health Economic Evaluation Reporting Standards 2022 (CHEERS 2022) Statement: Updated Reporting Guidance for Health Economic Evaluations. Value in Health 2022;25:3–9. https://doi.org/10.1016/j.jval.2021.11.1351.

[3] Vaughan K, Ozaltin A, Moi F, Kou Griffiths U, Mallow M, Brenzel L. Reporting gaps in immunization costing studies: Recommendations for improving the practice. Vaccine X 2020;5:100069. https://doi.org/10.1016/j.jvacx.2020.100069.

[4] Levin A, Boonstoppel L, Brenzel L, Griffiths U, Hutubessy R, Jit M, et al. WHO-led consensus statement on vaccine delivery costing: process, methods, and findings. BMC Medicine 2022;20:88. https://doi.org/10.1186/s12916-022-02278-4.

Table 4. Cost types and activities included in each of the reviewed studies.

| First author (year) | Country | Phase | Delivery strategy | Cost type | | | | | | | | | | | Activity | | | | | | | |
| --- | --- | --- | --- | --- | --- | --- | --- | --- | --- | --- | --- | --- | --- | --- | --- | --- | --- | --- | --- | --- | --- | --- |
|  |  |  |  | Per diems and allowances | Meeting or venue costs | Transportation and fuel costs | IEC materials and process | Vaccines | Vaccine supplies | Cold chain equipment | Capital cost of vehicles | Health worker time | Non health worker time | Other | Program planning and management | Training | Social mobilization and IEC | Vaccine collection or distribution and storage | Service delivery | Monitoring and evaluation | Supervision | Other |
| Alonso et al. (2019) | Mozambique | Demo | School-based | X (f) | X (f) | X (f) |  | X (o) | X (f) | X (o) | X (o) | X (o) | X (o) | Customs clearance (f) | X | X | X | X | X | X | X | Vaccine procurement |
| Soi et al. (2019)^1^ | Mozambique | Demo | School-based | X (f)^i^ | X (f)^i^ | X (f)^i^ | X (f)^i^ | X (f)^i^ | X (f)^i^ | X (o)^i^ |  | X (o)^i^ | X (o)^i^ | Overhead administrative costs (o)^i^ |  | X | X | X | X | X |  | Vaccine procurement |
| Levin et al. (2013)^2^ | Peru, Uganda, and Vietnam | Demo | School-based with facility-based and outreach, varying by country | X (f)^i^ | X(f)^i^ | X (f)^i^ |  |  | X (f)^i^ | X (o)^i^ | X (o)^i^ | X (o)^i^ |  |  | X | X | X | X | X |  |  | Waste management |
| Moodley et al. (2016)^3^ | South Africa | Demo | School-based |  | X(f)^i^ | X (f)^i^ | X(f)^i^ | X (o) | X (f)^i^ | X (f) ^i^ |  | X (o)^i^ |  | Printing (f)^i^ |  | X | X | X | X |  | X | Vaccine procurement, Waste management |
| Van Minh et al. (2017) | Vietnam | Demo | Facility-based ^i^ |  |  | X (f) | X (f) |  |  | X (f) | X (f) | X (o) |  | Maintenance costs (f) |  | X | X |  |  | X | X |  |
| Hidle et al. (2018) | Zimbabwe | Demo | School-based | X (f, o) | X (f, o) | X (f, o) | X (f, o) | X (o) | X (f) | X (o) | X (o) | X (o) |  | Communications (f, o) | X | X | X | X | X | X | X | Vaccination launch, Other meetings |
| Asfaw (2017)^4^ | Ethiopia | Pilot | School-based | X (f) | X (f)^i^ | X (f)^i^ | X (f) | X (o)^i^ | X (o)^i^ | X (o)^i^ |  | X (o) |  | Other equipment (o)^i^ | X | X | X | X | X | X | X | Waste management |
| Riewpaiboon et al. (2019) | Lao PDR | Pilot | School-based | X (f)^i^ | X (f)^i^ | X  (f)^i^ | X  (f)^i^ |  |  | X (f)^i^ |  | X (o) | X (o) |  | X | X | X | X | X | X | X | Vaccine procurement, Waste management |
| Quentin et al. (2012)^5^ | Tanzania | Pilot | School-based | X (f, o) | X | X (f, o) | X | X (o) | X | X (o)^i^ |  | X (f, o) | X | Customs clearance | X | X | X | X | X | X | X | Vaccine procurement, Waste management, Research |
| Ngabo et al. (2015)^6^ | Rwanda | Intro | School-based | X (f) | X (f) | X (f) | X (f) | X (f, o) | X (f) | X (o) |  | X (o) | X (o) | Customs clearance (f) | X | X | X | X | X | X | X | Waste management |
| Simuyemba et al. (2023)^7^ | Zambia | Intro | Mixed | X (f) | X (f) | X (f) | X (f) | X (f, o) | X (f) | X (f, o) | X (f) | X (f) |  | Printing costs (f), Other equipment (o)^i^, Building overhead (o) ^i^ | X | X | X | X | X | X | X | Waste management |
| Brennan et al. (2022) ^8^ | Senegal | Intro and routine | Mixed | X (f) | X (f, o) | X (f) ^i^ | X (f) ^i^ |  | X (f, o) | X (f, o)^i^ | X (f, o) ^i^ | X (o) | X (o) | Equipment and vehicle maintenance (f) ^i^, Customs clearance (f)^i^, Non-vaccination supplies and materials (f, o) | X | X | X | X | X | X | X | Vaccine procurement, Other activities |
| Hidle et al. (2022) ^8^ | Zimbabwe | Intro and routine | School-based | X (f) | X (f, o) | X (f) ^i^ | X (f) ^i^ |  |  | X (o)^i^ | X (f, o) ^i^ | X (o) | X (o) | Equipment and vehicle maintenance (f) ^i^, Non-vaccination supplies and materials (f, o) | X | X | X | X | X | X | X | Vaccine procurement, Other activities |
| Mvundura et al. | Ethiopia, Guyana, Rwanda, Senegal, Sri Lanka, and Uganda | Routine | School-based and mixed, varying by country | X (f) | X (f) | X (f) | X (f) |  |  | X (o) | X (o) | X (o) | X (o) | Shipping, handling and customs clearance (f), Printing/  Copying (f), Vehicle maintenance (f), Energy costs for cold storage (f), Fuel costs for incineration (f), Annualized cost of incinerators (o) | X | X | X | X | X | X | X | Vaccine procurement, Estimating demand, Crisis management, Waste management |
| Hsiao et al. (2023) | Tanzania | Routine | Mixed | X (f)^i^ | X (f)^i^ | X (f)^i^ |  | X (f, o) | X (f, o) | X (o) |  | X (o)^i^ | X (o)^i^ | Energy costs for cold storage (f)^i^, Annualized cost of incinerators (o)^i^, Non-vaccination supplies (f)^i^ | X | X | X | X | X |  | X |  |

| Abbreviations: f = financial cost, o = opportunity cost, i = inferred, X = cost type or activity included in scope of inputs, IEC = information, education, communication.  Note: Categorizations of cost types and activities based on main analysis and findings presented in each study. Due to the absence of uniformity across papers when describing the cost type or activities evaluated, the specific categorization used in the reviewed papers may not align precisely with the language of this table. We provide notes below for specific cases where categorizations were approximated.  Types of costs:  Financial costs (f) involve a direct financial outlay by the payor(s) while opportunity costs (o) represent the value of using existing resources [1]. Economic costs encompass the sum of financial and opportunity costs [1]. Differences in payor and perspective of the analysis may impact the classification of financial and economic costs. If a cost type was not specified as a financial or opportunity/economic cost in the paper, we made inferences where possible and denoted the classification with an “i”. In some cases, we did not find there was enough information to make an inference and so left the cost type unmarked.  Definitions of cost types included in the HPV vaccine program costs, with shared resources allocated to HPV vaccine program as relevant:  *Per diems and allowances: “*Any allowances paid or paid to volunteer workers for immunization-related activities” [1].  *Meeting or venue costs:* “Space rented for a particular activity, such as hotel or meeting room for training or meetings, or office space” and any related audiovisual equipment, catering, and meeting costs. Adapted from [2].  *Transportation and fuel costs:* Cost of public transportation, hired vehicles, plane travel, and fuel for immunization-related transport. Adapted from [1].  *IEC materials and process:* Cost of printing IEC materials and producing, distributing, or airing other media such as radio messages. Adapted from [1].  *Vaccines*: Cost of vaccine product. Opportunity costs denote the value of subsidized or donated vaccines, while financial cost indicates health system payment for vaccines. Adapted from [1].  *Vaccine supplies*: Cost of syringes, safety boxes, and other dry supplies used for administration of vaccines. Adapted from [1].  *Cold chain equipment:* “Value of all cold chain equipment” or space “used to store and transport vaccines.” Adapted from [1].  *Capital cost of vehicles:* “Value of all vehicles and modes of transport” used for the vaccine program activities, may include boats, ATVs, and motorcycles. Adapted from [1].  *Health worker time:* Time costs of immunization program staff.  *Non health worker time:* Time costs of volunteers, ministry of education/school staff, and community stakeholders.  *Other cost categories:* Other cost types for the vaccination program, including other equipment (incinerators) and capital, overhead costs, energy costs for cold storage and/or waste management, vehicle and equipment maintenance, non-vaccination supplies, and the cost of customs clearance for vaccines. Adapted from [1].  Definitions of activities costed for the HPV vaccine program:  *Program planning and management*: “Time and resources spent on planning, budgeting, and managing the immunization program at various levels,” including microplanning. Adapted from [1].  *Training*: “Time and resources spent attending and/or providing immunization-related training” [1].  *Social mobilization and IEC*: “Social mobilization includes holding community meetings, printing flyers and educational materials, conducting events, and other sensitization of the community” [1].  *Vaccine collection or distribution and storage*: “Time and resources spent collecting vaccines at the airport or other distribution points, storing vaccines in national or subnational cold stores” and health facilities, “maintaining stock records of vaccines, and distributing vaccines down to the facility.” Adapted from [1].  *Service delivery*: “Time and resources spent on the act of administering the vaccine to children” at the health facility, in schools, and at outreach locations. Adapted from [1].  *Monitoring and evaluation*: “Time and resources spent on data entry and analysis, maintaining records of children vaccinated, completing reports and analysis, and monitoring and evaluating immunization program data.” Adapted from [1].  *Supervision*: “Time and resources spent by facility” (national or subnational administrative program) “staff to supervise subordinate or peer health or community workers.” Adapted from [1].  *Other activities*: Other activities that are part of the vaccination program including crisis management (surveillance), and other activities not mentioned above. Adapted from [1].  Notes on specific categorizations:  ^1^ We categorized “storage costs” and “use of cold chain excess capacity and capital costs of existing equipment” as cold chain equipment and capital cost of vehicles. We also identified the activity vaccine collection or distribution and storage for what the Soi et al. describe as “cold chain supplement.”  ^2^ We assumed that meeting and venue costs were included as part of “all expenses associated with training workshops.”  ^3^ We assumed that meeting and venue costs were included as part of “costs of training.” Cold chain equipment refers to cold boxes and additional refrigerators only, as “capital costs for equipment already in hospital/pharmacy was not included.” We also determined that “Administration” in the context of the paper referred to the administration of HPV vaccines, and therefore categorized this as service delivery.  ^4^ Based on the activities described under “vaccines and supplies” in the paper, the systematic reviewers inferred that this activity also encompassed vaccine collection or distribution and storage. We assumed that meeting and venue costs were included as part of training costs.  ^5^ We determined that the activity of “preparation” corresponded best with program planning and management. Similarly, based on the described activities under “administration/supervision”, which included data management and project coordination, we have categorized this as program planning and management and monitoring and evaluation.  ^6^ Vaccine collection or distribution and storage activity inferred based on inclusion of cold chain costs and costs to store HPV vaccines.  ^7^ Vaccine supplies cost category does not include syringes, as text references only “waste management supplies bought specifically for HPV vaccine.”  ^8^ Vaccine supplies cost category does not include syringes or safety boxes, as text in notes: “Vaccination supplies refers to other supplies, such as cotton, that were used as part of vaccination.” |
| --- |

Supplemental Table 4 References

[1] Resch S, Menzies N, Portnoy A, Clarke-Deelder E, O’Keeffe L, Suharlim C, et al. How to cost immunization programs: a practical guide on primary data collection and analysis 2020. Available at: <https://immunizationeconomics.org/recent-activity/2019howtocost>.

[2] Brennan T, Hidle A, Doshi RH, An Q, Loharikar A, Casey R, et al. Cost of human papillomavirus vaccine delivery in a single-age cohort, routine-based vaccination program in Senegal. Vaccine 2022;40:A77–84. <https://doi.org/10.1016/j.vaccine.2021.11.057>.

Table 5. Extracted unit cost estimates for delivery costs, excluding vaccine supplies and procurement costs, in 2022 US$.

| **First author (year)** | **Country** | **Phase** | **Study subgroup,**  **if reported** | **Currency year for costs** | **Cost estimates (excluding vaccine cost)**  **if reported** | | | |
| --- | --- | --- | --- | --- | --- | --- | --- | --- |
|  |  |  |  |  | **Financial cost per dose** | **Economic cost per dose** | **Financial cost per FIC** | **Economic cost per FIC** |
| Alonso et al. (2019) | Mozambique | Demo | Full study | 2014 | $5.41 |  | $16.00 |  |
| Levin et al. (2013) | Peru, Uganda, and Vietnam | Demo | Peru school-based | 2009 | $2.58 | $4.94 |  |  |
|  |  |  | Uganda school-based | 2009 | $2.29 | $3.44 |  |  |
|  |  |  | Uganda integrated outreach | 2009 | $1.21 | $1.57 |  |  |
|  |  |  | Vietnam school-based | 2009 | $2.38 | $2.38 |  |  |
|  |  |  | Vietnam health center-based | 2009 | $2.28 | $2.82 |  |  |
| Van Minh et al. (2017) | Vietnam | Demo | Full study | 2012* |  |  | $12.75 |  |
| Hidle et al. (2018) | Zimbabwe | Demo | Full study | 2016 | $24.07 | $48.70 | $48.77 | $98.68 |
| Asfaw (2017) | Ethiopia | Pilot | Ahefrom district | 2016* |  |  | $14.28 | $26.61 |
|  |  |  | Gomma district | 2016* |  |  | $6.24 | $11.21 |
| Riewpaiboon et al. (2019) | Lao PDR | Pilot | 3-dose schedule | 2013 | $2.24 | $2.85 | $6.73 | $8.54 |
|  |  |  | 2-dose schedule | 2013 | $2.44 | $3.38 | $4.87 | $6.77 |
| Ngabo et al. (2015) | Rwanda | Intro | Full study | 2012 | $3.41 | $4.82 | $10.37 | $14.64 |
| Brennan et al. (2022) | Senegal | Intro and routine | Full study | 2020 | $3.32 | $8.17 |  |  |
| Hidle et al. (2022) | Zimbabwe | Intro and routine | Full study | 2020 | $0.60 | $1.48 |  |  |
| Mvundura et al. (2023) | Ethiopia, Guyana, Rwanda, Senegal, Sri Lanka, and Uganda | Routine | Ethiopia | 2019 | $2.55 | $8.23 |  |  |
|  |  |  | Guyana | 2019 | $2.36 | $19.36 |  |  |
|  |  |  | Rwanda | 2019 | $1.16 | $3.47 |  |  |
|  |  |  | Senegal | 2019 | $3.53 | $12.89 |  |  |
|  |  |  | Sri Lanka | 2019 | $0.31 | $4.44 |  |  |
|  |  |  | Uganda | 2019 | $3.77 | $8.61 |  |  |

Abbreviations: demo = demonstration, FIC = fully immunized child, intro = introduction

*Inferred by systematic reviewers.
